# Effects of the CB1 receptor antagonists AM6545 and AM4113 on metabolic syndrome-induced prostatic hyperplasia in rats

**DOI:** 10.17305/bb.2023.9173

**Published:** 2023-12-01

**Authors:** Basma G Eid, Thikryat Neamatallah, Lenah S Binmahfouz, Amina M Bagher, Abdulmohsin J Alamoudi, Hibah Mubarak Aldawsari, Abeer Hanafy, Atif Hasan, Hany M El-Bassossy, Ashraf B Abdel-Naim, Kiran Vemuri, Alexandros Makriyannis

**Affiliations:** 1 Department of Pharmacology and Toxicology, Faculty of Pharmacy, King Abdulaziz University, Jeddah, Saudi Arabia; 2 Department of Pharmaceutics, Faculty of Pharmacy, King Abdulaziz University, Jeddah, Saudi Arabia; 3 Department of Pharmacology, Faculty of Veterinary Medicine, Kafrelsheikh University, Kafrelsheikh, Egypt; 4 Department of Anatomy and Embryology, Faculty of Veterinary Medicine, Kafrelsheikh University, Kafrelsheikh, Egypt; 5 Department of Pharmacology and Toxicology, Faculty of Pharmacy, Zagazig University, Zagazig, Egypt; 6 Center for Drug Discovery, Northeastern University, Boston, MA, USA; 7 Departments of Chemistry and Chemical Biology and Pharmaceutical Sciences, Northeastern University, Boston, MA, USA

**Keywords:** AM6545, AM4113, prostate, cannabinoid antagonist, metabolic syndrome (MetS).

## Abstract

Metabolic syndrome (MetS) is a combination of metabolic disorders that can predispose individuals to benign prostatic hyperplasia (BPH). The inhibition of the cannabinoid 1 (CB1) receptor has been used to treat metabolic disorders in animal models. This study reports the use of a peripherally restricted CB1 antagonist (AM6545) and a neutral CB1 antagonist (AM4113) to improve MetS-related BPH in rats. Animals were divided into three control groups to receive either a normal rodent diet, AM6545, or AM4113. MetS was induced in the fourth, fifth, and sixth groups using a concentrated fructose solution and high-salt diet delivered as food pellets for eight weeks. The fifth and sixth groups were further given AM6545 or AM4113 for additional four weeks. Body and prostate weights were measured and prostate sections were stained with hematoxylin eosin. Cyclin D1, markers of oxidative stress and inflammation, and levels of the endocannabinoids were recorded. BPH in rats with MetS was confirmed through increased prostate weight and index, as well as histopathology. Treatment with either AM6545 or AM4113 significantly decreased prostate weight, improved prostate histology, and reduced cyclin D1 expression compared with the MetS group. Groups treated with CB1 antagonists experienced reduced lipid peroxidation, recovered glutathione depletion, restored catalase activity, and had lower inflammatory markers interleukin 6 (IL-6) and tumor necrosis factor alpha (TNF-α). MetS rats treated with either AM6545 or AM4113 showed reduced concentrations of anandamide (AEA) and 2-arachidonoylglycerol (2-AG) in the prostate compared with the MetS group. In conclusion, the CB1 antagonists AM6545 and AM4113 protect against MetS-induced BPH through their anti-proliferative, antioxidant, and anti-inflammatory effects.

## Introduction

Benign prostatic hyperplasia (BPH) is a common histologic finding in aging men and refers to nonmalignant development or hyperplasia of prostatic tissue [1]. It is an important cause of lower urinary tract symptoms, such as weak urinary stream, impaired bladder emptying, post-void dribbling, and the sensation of incomplete bladder emptying after voiding [2]. As reported by the American Urological Association, the prevalence of BPH is over 50% in men over 60 years of age and increases to 90% in men over 80 years of age [3]. Although the mechanism of BPH development remains unclear, multiple risk factors have been suggested, such as aging, inflammation, direct infection, and oxidative stress [2]. In addition, recent studies have demonstrated a clear association between BPH and metabolic syndrome (MetS) [4–7].

MetS is a combination of metabolic abnormalities that include central obesity, dyslipidemia, insulin resistance, and primary hypertension [8]. Similar to BPH, the prevalence of MetS steadily increases with age [9]. Xu et al. [10] have shown that metabolic disorders, including obesity and insulin resistance, can promote the development of BPH in rats consuming a high-fat diet. Another study demonstrated that the prevalence of BPH is higher in people with MetS compared with those without MetS [6]. This has been shown to be due to insulin-related stimulation of prostate growth, induction of systemic inflammation and oxidative stress, and sex hormone alterations [6].

The endocannabinoid system (ECS) plays a crucial role in various physiological and pathological conditions, such as analgesia, inflammation, cancer, MetS, and others. The ECS consists of the human cannabinoid 1 receptor (CB1) and cannabinoid 2 receptor (CB2), endogenous lipid-signaling molecules (endocannabinoids), primarily 2-arachidonoylglycerol (2-AG) and anandamide (AEA), as well as the enzymes that synthesize and degrade endocannabinoids. While CB1 is abundantly expressed in the central nervous system, CB2 is found mainly in immune cells [11, 12]. In addition, CB1 is peripherally expressed in several tissues, such as the human prostate. Specifically, CB1 is present on the human prostate epithelial layer and the sensory nerves that innervate the human prostatic stroma [13, 14]. Furthermore, the human prostate expresses the enzyme fatty acid amidohydrolase, which hydrolyzes AEA [13]. The expression of ECS components in prostatic tissue indicates that this system may influence normal prostate physiology. It has been suggested that the ECS may play a role in the pathogenesis of BPH [15]. One study suggested that antagonism of the CB1 receptor with rimonabant (SR141716) may be a useful and effective method for the prevention and treatment of BPH by reducing the size of a noncancerous hypertrophied prostate [16]. Therefore, more research is needed to fully understand the important role of ECS blockade using different modulators in the prevention and treatment of BPH.

The correlation between MetS and BPH has offered new potential targets for the treatment and prevention of both conditions. In this regard, selective inhibition of CB1 has shown promising therapeutic results in obesity-related metabolic disorders [17]. Rimonabant was the first CB1-selective antagonist/inverse agonist approved for the treatment of obesity and MetS [18]. However, in 2008, rimonabant was withdrawn due to adverse psychiatric side effects, including suicide attempts, anxiety, and depression, resulting from CB1 antagonism in the central nervous system (CNS) [19]. To prevent the adverse effects of CB1 antagonism in the CNS, different strategies to design a peripherally restricted CB1 antagonist (AM6545) and a neutral CB1 antagonist (AM4113) have been developed in this laboratory [20–24]. We recently showed that AM6545 and AM4113 attenuated peripheral IR in rats with MetS. Additionally, they exerted anti-hyperuricemia, anti-dyslipidemia, and anti-inflammatory effects [25, 26].

Therefore, the current study aimed to investigate whether peripheral blockade of CB1 might be useful in preventing MetS-related BPH. For that, a concentrated 20% w/v fructose solution and a high-salt diet as food pellets were used for eight weeks to establish a rat model of MetS associated with BPH. The CB1 antagonists AM6545 and AM4113 were administered to MetS rats to examine the effect of CB1 blockage on prostate structure and function.

## Materials and methods

### Chemicals

The CB1 antagonists AM4113 (5-(4-alkylphenyl)-1-(2,4-dichlorophenyl)-4-methyl-N-(piperidin-1-yl)-1H-pyrazole-3-carboxamide) and AM6545 (5-(4-[4-cyanobut-1-ynyl]phenyl)-1-(2,4-dichlorophenyl)-4-methyl-N-(1,1-dioxo-thiomorpholino)-1H-pyrazole-3-carboxamide) were synthesized in our laboratory at the Center for Drug Discovery at Northeastern College in Boston, MA, USA, as described previously [20, 27]. Fructose and sodium chloride salt were purchased from Sigma-Aldrich (St. Louis, MO, USA). AM6545 is a peripherally acting CB1 neutral antagonist (the first neutral peripherally acting neutral antagonist). On the other hand, AM4113 is also a neutral antagonist, but unlike AM6545, it enters the brain. AM4113 is more lipid soluble and therefore should have better penetration into prostate tissue than AM6545.

### Animals

Forty-eight male Wistar rats (45 days old and 150–190 g body weight) were procured from King Abdulaziz University (KSA) to model the fructose-induced MetS as in our previous published work [25]. There were no significant differences in the baseline body weights of the animals. Rats were housed in a temperature-controlled room (23 ± 1 ^∘^C) with a 12-h light/dark cycle. Prior to the experiment, animals were fed standard rat chow ad libitum and had unlimited access to water for seven days.

### Metabolic syndrome model and study design

Rodents were divided into six groups of eight animals each, as previously reported [25]. Group I (control) was fed standard rodent food and had free access to water. Groups II and III were control groups that were administered AM6545 (A65 group) or AM4113 (A41 group) drugs, respectively. Group IV (MetS group) was fed standard rodent food but had free access to a 20% w/v fructose solution and food pellets with 3% salt for 8 consecutive weeks. Groups V and VI were MetS groups that were treated with AM6545 (MetS + A65 group) or AM4113 (MetS + A41 group) drugs for an additional four weeks, while groups I–IV continued to receive the same diet. Both compounds were administered intraperitoneally (IP) to groups II, III, V, and VI as 0.5% carboxymethyl cellulose (CMC) suspension at a dose of 10 mg/kg/day (dosing volume 10 mL/kg), whereas control group and the MetS groups received only CMC.

After 12 weeks, rats were anesthetized with urethane (1 g/kg IP) [28]. The ventral prostates were excised and weighed. A portion of the ventral prostate was rapidly removed from each animal and immediately stored at −80 ^∘^C for biochemical measurements. A different part of the ventral prostate was kept in 10% formalin for histological examination. The development of MetS was proven by increased body weight, visceral fat accumulation, dyslipidemia, enhanced homeostasis model assessment-estimated insulin resistance (HOMA-IR), and increased mean arterial blood pressure, as reported in our previous study [25].

### Body and prostate weight

After dissection, the weight of the prostate of each rat was recorded. Relative prostate weight was calculated by dividing the prostate weight (g) by the body weight of the rat (g) and then multiplying by 10^3^ [29].

### Prostate homogenates

Prostate specimens were homogenized by the ULTRA-TURRAX^®^ homogenizer using a homogenization buffer (1% Triton X 100 + phosphate buffer saline, pH 7.4). Centrifugation of the homogenates for 20 min at 4000× and 4 ^∘^C was then performed. The supernatant was collected and kept at —80 ^∘^C for subsequent analysis [30].

### Histological examination of the prostate

To prepare for sectioning, formalin-fixed prostate samples were routinely processed and embedded in paraffin blocks. Five micrometer (5 µm) thick paraffin sections were obtained for staining. Hematoxylin and eosin (H&E) staining was used to examine histological architecture. At least three of the stained sections from each rat were examined by electric light microscopy (Nikon Eclipse TE2000-U, NIKON, Japan), and the height of the prostate glandular epithelium was determined by Image J software (1.46a, NIH, USA).

### Immunohistochemical staining for cyclin D1 detection

Immunohistochemical detection of the proliferation marker cyclin D1 was performed on prostate sections kept in paraffin. The tissue sections were dried, deparaffinized, and rehydrated in ethanol. The sections were then boiled for 5 min in a 10 mM sodium citrate buffer (pH 6.0) for 5 min, washed with phosphate-buffered saline, and immunostained. Afterward, the sections were kept for 2 h at room temperature in 5% bovine serum albumin (BSA) in tris-buffered saline (TBS). This was followed by overnight incubation with 1 µg/mL rabbit polyclonal anti-cyclin D1 primary antibody at 4 ^∘^C (ABCAM, Cambridge, UK). Slides were washed the next day with TBS before being kept with the biotinylated secondary antibody using the Cell and Tissue Staining Kit (R&D Systems, MN, USA). A light microscope was used to examine the slides. Photomicrographs were taken with a CCD camera and quantified using ImageJ software (ImageJ, 1.46a, NIH, USA) [31].

### Measurement of oxidative stress markers

According to the manufacturer’s instructions, kits purchased from Biodiagnostics (Cairo, Egypt) were used to determine the concentrations of malondialdehyde (MDA, product no. MD-252), superoxide dismutase (SOD, product no. SD-252), and catalase (CAT, product no. CA-2516) in prostate homogenates.

### Endocannabinoid measurements

According to the manufacturer’s instructions, Real-Gene Labs (Lake Forest, CA, USA) kits were used to measure endocannabinoids AEA and 2-AG in prostate homogenates.

### Measurements of inflammatory markers

An enzyme-linked immunosorbent assay (ELISA) was performed to measure the concentrations of interleukin 6 (IL-6) and tumor necrosis factor alpha (TNF-α) in prostate tissue homogenates (ab234570, ab100785, ABCAM UK, respectively). Quantification was performed using an automated ELISA reader.

### Ethical statement

All experimental animal procedures were performed at King Abdulaziz University according to the “Guidelines for Animal Care and Treatment of the Biomedical Ethics Research Committee” (Reference No. 479-16). Every effort was made to keep animal suffering to a minimum and reduce the number of animals used.

### Statistical analysis

All data are presented as means ± standard deviation (S.D.). GraphPad Prism (Version 9) was used to analyze the different groups using one-way analysis of variance (ANOVA) and Tukey’s post hoc test for multiple comparisons. *P* < 0.05 was used to indicate statistical significance.

## Results

### Body and prostate weight

Treatment of rats with AM6545 (A65 group) or AM4113 (A41 group) alone did not adversely affect prostate weights (*P* > 0.05) relative to the controls (Table 1). However, fructose solution and high-salt food pellets fed (MetS group) rats significantly increased relative prostate weight by 67% compared with controls (*P* < 0.05). Importantly, administration of AM6545 (MetS + A65 group) or AM4113 (MetS + A41 group) to MetS rats significantly decreased relative prostate weight by approximately 26% or 30%, respectively (*P* < 0.05).

**Table 1:** Effect of AM6545 and AM4113 on prostate weight in MetS-induced BPH in rats

	**Prostate weight (g)**	**Body weight (g)**	**Relative prostate weight (×10^3^)**
Control	0.25 ± 18.3	260.3 ± 22.1	0.96 ± 0.07
A65	0.24 ± 20.1	254.0 ± 21.7	0.94 ± 0.12
A41	0.27 ± 19.5	259.3 ± 20.8	1.04 ± 0.01
MetS	0.64 ± 55.3	380.1 ± 31.1	1.68 ± 0.02^a,b,c^
MetS + A65	0.42 ± 33.5	343.4 ± 28.2	1.22 ± 0.04^a,b,c,d^
MetS + A41	0.37 ± 30.1	325.3 ± 27.1	1.13 ± 0.18^a,b,d^

Results are displayed as mean ± S.D. (*n* ═ 8). Statistical significance is shown when *P* < 0.05 by one-way ANOVA with Tukey’s post hoc test. a, b, c, or d indicates a significant difference from the control, A65, A41, or MetS groups, respectively. A65 is the AM6545-treated group, A41 is the AM4113-treated group, MetS is the metabolic syndrome group, and MetS + A65 or MetS+ A41 groups are MetS groups treated with AM6545 or AM4113, respectively. MetS: Metabolic syndrome; BPH: Benign prostatic hyperplasia; ANOVA: Analysis of variance.

### Histological examination of the prostate

**Figure 1.**
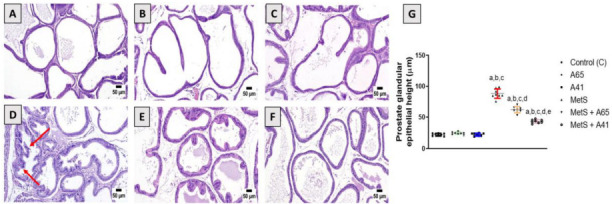
**Impact of AM6545 and AM4113 treatment on prostate histopathology in MetS-induced BPH in rats.** The MetS rats were treated with 10 mg/kg AM6545 or AM4113 daily for four weeks. Prostate sections stained with hematoxylin and eosin (H&E) are shown. (A) Control group showing the normal prostate histoarchitecture; (B) AM6545 (A65) and (C) AM4113 (A41) alone groups showed no observable histological changes; (D) MetS group showing hyperplasia with increased epithelial thickness; (E) MetS + AM6545 (MetS + A65) and (F) MetS + AM4113-treated (MetS + A41) groups showing a clear reduction in hyperplasia and projections; (G) Graphical display of prostate glandular epithelium height. Results are shown as mean ± S.D. (*n* ═ 8). *P* < 0.05 was considered statistically significant by one-way ANOVA with Tukey’s post hoc test. a, b, c, d, or e indicates a significant difference from the control, A65, A41, MetS, or MetS + A65 groups, respectively, at *P* < 0.05 employing one-way ANOVA followed by Tukey’s post hoc test. MetS: Metabolic syndrome; BPH: Benign prostatic hyperplasia; ANOVA: Analysis of variance; S.D.: Standard deviation.

Histopathology of the control group (Figure 1A) and the groups that received AM6545 (Figure 1B) and AM4113 (Figure 1C) showed normal histoarchitecture with well-defined glandular structures, single-layered epithelial cells lining the ductules, well-differentiated stroma and well-defined interductal spaces. After eight weeks of sustained fructose and salt feeding, marked histological changes occurred in the prostatic ductules and interstitial tissue in the MetS group. The prostatic ductules showed dilated lumens, irregular boundaries, enlarged luminal epithelium, and scanty secretion (Figure 1D). The interductal spaces became wider and filled with interstitial tissue. The luminal epithelium was greatly increased in height (Figure S1). Treatment of rats with AM6545 and AM4113 (Figure 1E and 1F) induced a dramatic improvement in the histologic profile of the prostate, which was almost similar to the control ones. Prostatic ductules restored their normal outline. The luminal epithelium had normal heights, and marked epithelial folds protruded into the ductal lumens. The interductal spaces were greatly reduced reaching normal spacing; the prostatic stroma appeared crowded with ductules and interductal spaces. The height of the luminal epithelium in the different experimental groups is shown in Figure 1G. The groups treated with AM6545 or AM4113 did not show any changes compared with the control group. However, there was a marked increase in epithelial heights by 300% in the MetS group relative to controls. The epithelial heights in MetS rats cotreated with AM6545 and AM4113 were significantly decreased by 30% and 52%, respectively, compared with MetS rats.

### Cyclin D1 assessment as a proliferation marker

**Figure 2.**
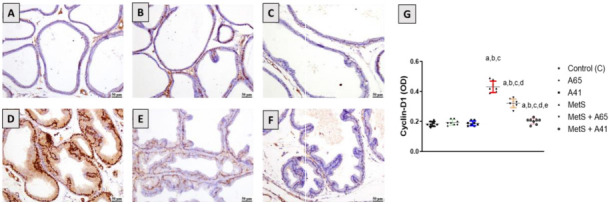
**Photomicrographs showing the effect of AM6545 or AM4113 treatment on the prostate expression of cyclin D1 in MetS-induced BPH in rats.** The MetS rats were treated with 10 mg/kg AM6545 or AM4113 daily for 4 weeks. The concentration of cyclin D1 expression was determined by immunohistochemistry. (A) Control group; (B) AM6545-treated group (A65); (C) AM4113-treated group (A41); (D) MetS group; (E) MetS rats cotreated with AM6545 (MetS + A65); (F) MetS rats cotreated with AM4113 (MetS + A41); (G) Quantification of cyclin D1 expression in the prostate in all groups. Scale bar (50 µm). Results are displayed as mean ± SD (*n* ═ 8). a, b, c, d, or e indicates a significant difference from the control, A65, A41, MetS, or MetS+A65 groups, respectively, at *P* < 0.05 employing one-way ANOVA followed by Tukey’s post hoc test. MetS: Metabolic syndrome; BPH: Benign prostatic hyperplasia; ANOVA: Analysis of variance.

Immunohistochemical analysis of the expression of cyclin D1 protein as a proliferation marker revealed a moderate amount of stained cells in the control groups (Figure 2A–2C). On the other hand, an increase in stained cells was observed in the MetS group, indicating an increase in the proliferation rate (Figure 2D). Treatment of MetS rats with AM6545 or AM4113 significantly reduced cyclin D1-positive cells compared with MetS rats (Figure 2E and 2F). Densitometric analysis of cyclin D1-positive cells indicated that AM6545 and AM4113 reduced cyclin D1 expression by 26% and 54%, respectively (Figure 2G).

**Figure 3.**
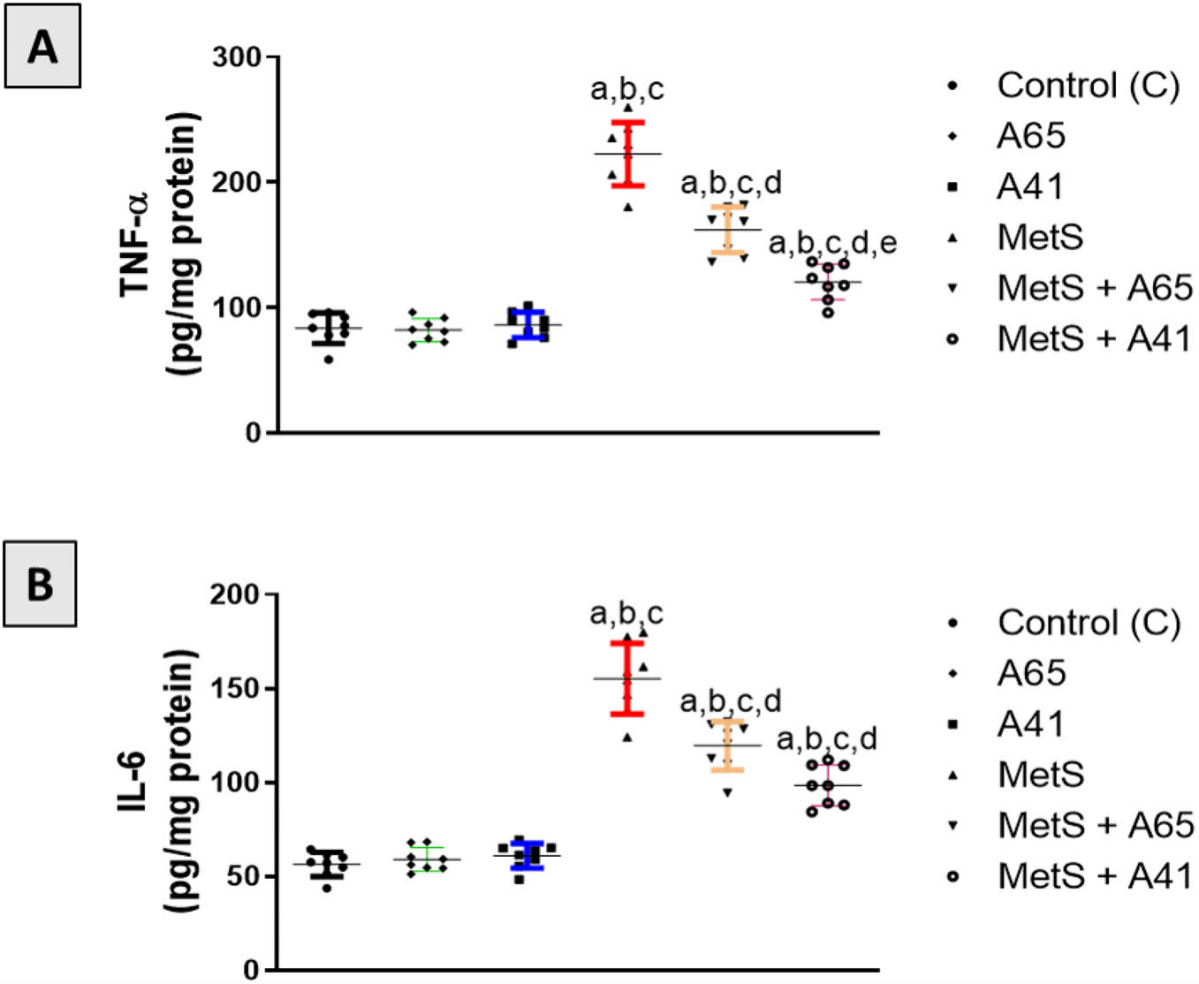
**Effect of AM6545 or AM4113 treatment on prostate content of TNF*-α*** **and IL-6 in MetS-induced BPH in rats.** The MetS rats were treated with 10 mg/kg AM6545 or AM4113 daily for four weeks. The concentration of (A) TNF*-α* and (B) IL-6 were determined in the prostate tissue. A65 is the AM6545-treated group, A41 is the AM4113-treated group, MetS is the metabolic syndrome group, and MetS + A65 or MetS+ A41 groups are MetS groups treated with AM6545 or AM4113, respectively Data are expressed as mean ± S.D. (*n* ═ 8). a, b, c, d, or e indicates a significant difference from the control, A65, A41, MetS, or MetS+A65 groups, respectively, at *P* < 0.05 using one-way ANOVA followed by Tukey’s post hoc test. TNF*-α*: Tumor necrosis factor alpha; IL-6: Interleukin 6; MetS: Metabolic syndrome; BPH: Benign prostatic hyperplasia; ANOVA: Analysis of variance; S.D.: Standard deviation.

**Table 2:** Effect of AM6545 and AM4113 on oxidative stress in a BPH rat model of MetS

	**MDA (nmol/mg protein)**	**SOD (U/mg protein)**	**CAT (U/mg protein)**
Control	0.39 ± 0.04	12.1 ± 0.9	66.4 ± 5.2
A65	0.40 ± 0.05	11.9 ± 1.0	62.8 ± 5.4
A41	0.41 ± 0.05	12.4 ± 1.1	63.2 ± 5.8
MetS	3.26 ± 0.31^a,b,c^	7.3 ± 0.6^a,b,c^	44.7 ± 4.1^a,b,c^
MetS + A65	2.21 ± 0.23^a,b,c,d^	9.8 ± 0.9^a,b,c,d^	65.6 ± 5.3^d^
MetS + A41	1.07 ± 0.11^a,b,c,d,e^	10.3 ± 1.1^a,b,c,d^	65.3 ± 4.8^d^

Results are shown as mean ± S.D. (*n* ═ 8). Results are shown as mean ± S.D. (*n* ═ 8). *P* < 0.05 was considered statistically significant by one-way ANOVA with Tukey’s post hoc test. a, b, c, d, or e indicates a significant difference from the control, A65, A41, MetS, or MetS+A65 groups, respectively. A65 is an AM6545-treated group, A41 is an AM4113-treated group, MetS is a metabolic syndrome group, and MetS + A65 or + A41 groups are MetS groups treated with AM6545 or AM4113, respectively. MDA: Malondialdehyde, SOD: Superoxide dismutase, CAT: Catalase; BPH: Benign prostatic hyperplasia; MetS: Metabolic syndrome; S.D.: Standard deviation.

### Assessment of prostate oxidative stress markers

To assess the effects of AM6545 and AM4113 treatments on oxidative stress in the prostate, measurements of MDA as a lipid peroxidation marker as well as SOD and CAT activities in prostatic tissues were performed. The CB1 antagonist groups (A65 or A41 groups) did not cause changes in the measured oxidative stress markers (Table 2). On the other hand, MetS rats showed a significant increase in the concentration of MDA in prostate tissue by 736% compared with controls. Administration of AM6545 (MetS + A65 group) or AM4113 (MetS + A41 group) to MetS rats caused a marked lowering of MDA concentrations by 33% and 68%, respectively, compared with MetS rats. Post hoc analysis demonstrated that MDA reduction in the MetS + A41 group was superior to that in the MetS + A65 group (*P* < 0.05). However, the activities of the antioxidant enzymes SOD and CAT were decreased by 40% and 33%, respectively, in the prostate tissue of the MetS rats compared with the control group. Administration of AM6545 or AM4113 markedly ameliorated the reduction of SOD activity and increased it by 34% after AM6545 and by 41% after AM4113 relative to the MetS group. Similarly, CAT activity increased by 46% upon treatment with both CB1 antagonists. Therefore, the antioxidant activity of AM6545 and AM4113 might be responsible for their protective effects on MetS-induced BPH in rats.

### Assessment of TNF-α and IL-6 in prostatic tissue

The anti-inflammatory activity of the CB1 antagonists AM6545 and AM4113 in MetS-induced BPH was explored. Controls had low concentrations of TNF-α and IL-6 (Figure 3). Treatment of control rats with AM6545 or AM4113 alone did not change the concentration of these inflammatory markers. However, TNF-α and IL-6 levels were significantly increased by 167% and 176%, respectively, in the prostate tissues of MetS rats relative to controls. Treatment of MetS rats with AM6545 or AM4113 (10 mg/kg) significantly decreased TNF-α by 24% and 46%, respectively, and IL-6 by 24% and 37%, respectively (*P* < 0.05).

### Measurement of the prostate content of anandamide (AEA) and 2-arachidonoyl glycerol (2-AG)

**Figure 4.**
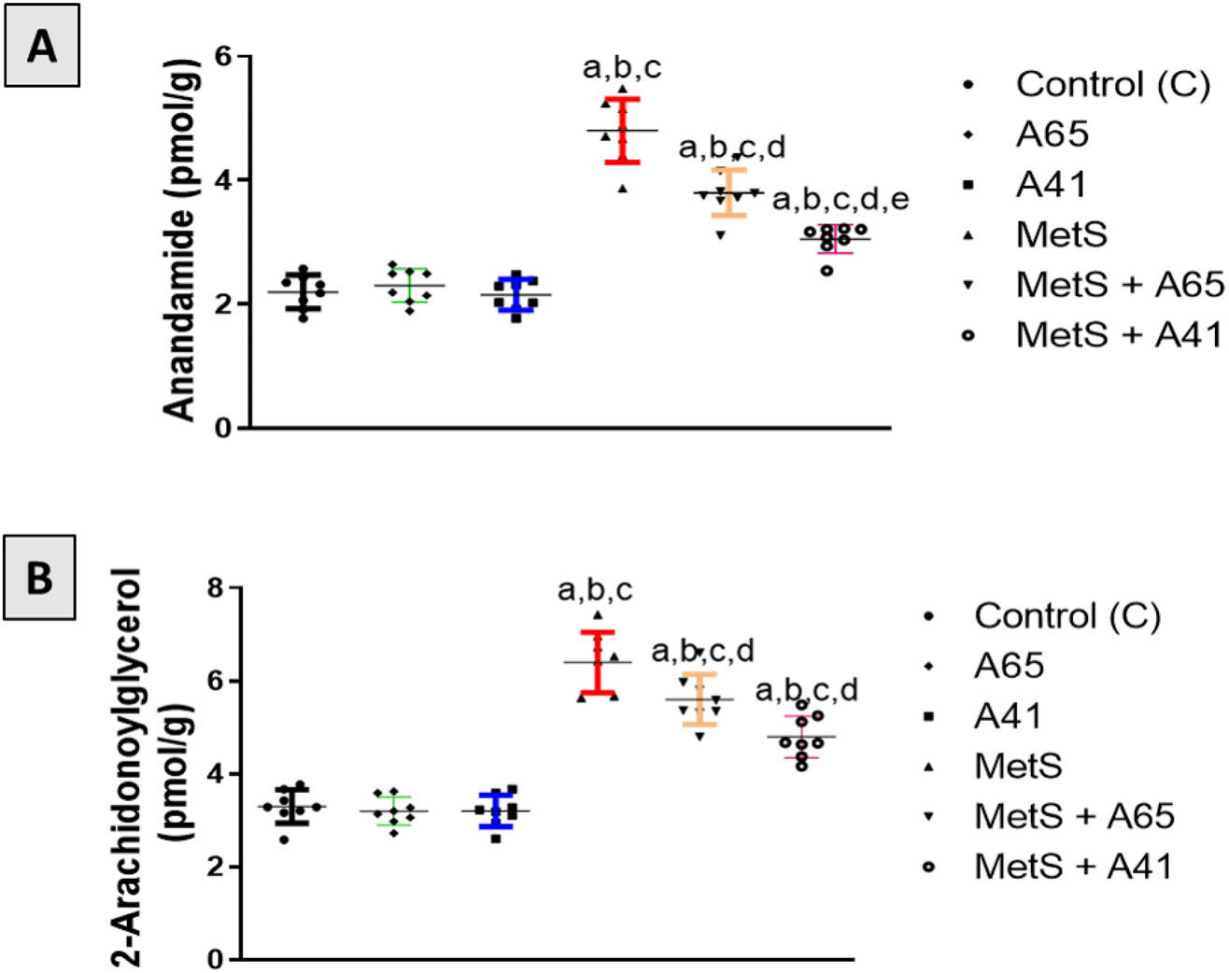
**Effect of AM6545 and AM4113 on anandamide (AEA) and 2-arachidonoyl glycerol (2-AG) contents in the prostate of MetS-induced BPH in rats.** The MetS rats were treated with 10 mg/kg AM6545 or AM4113 daily for four weeks. The concentrations of (A) anandamide and (B) 2-AG were determined in the prostate tissue. A65 is the AM6545-treated group, A41 is the AM4113-treated group, MetS is the metabolic syndrome group, and MetS + A65 or MetS+ A41 groups are MetS groups treated with AM6545 or AM4113, respectively Bars are mean ± S.D. (*n* ═ 8). a, b, c, or d indicates a significant difference from the control, A65, A41, or MetS groups, respectively, at *P* < 0.05 one-way analysis of variance (ANOVA) followed by the Bonferroni test for multiple comparisons. MetS: Metabolic syndrome; BPH: Benign prostatic hyperplasia; S.D.: Standard deviation.

To assess endocannabinoid activity in the prostate of MetS rats, concentrations of the endogenous cannabinoids AEA and 2-AG were measured in prostate homogenates. No change in the concentration of prostatic AEA and 2-AG was observed in control rats treated with either AM6545 (A65 group) or AM4113 (A41 group) (Figure 4). However, AEA and 2-AG contents were markedly increased in the prostates of MetS rats by 118% and 93%, respectively, compared with the controls. Treatment of MetS rats with AM6545 (MetS + A65 group) or AM4113 (MetS + A41 group) significantly reduced the concentration of AEA by 21% and 38% and of 2-AG by 13% and 25% in the prostate relative to the MetS group. Post hoc analysis demonstrated that the reduction of AEA was more significant in animals in the MetS + A41 group than in the MetS + A65 group (*P* < 0.05).

## Discussion

BPH is a common medical condition that affects men as they age and is strongly associated with MetS. This noncancerous enlargement of the prostate gland significantly compromises the outflow of urine from the bladder, leading to several symptoms such as urinary retention. Increased expression of CB1 has been found in BPH and prostate cancer tissue, highlighting the therapeutic potential of targeting this receptor in these diseases [32–34]. However, CB1 is also highly expressed in the CNS. Hence, targeting this receptor has been associated with severe psychiatric side effects, including depression, anxiety, and suicide attempts [19]. Selective targeting of CB1 in the periphery is a promising therapeutic strategy for managing BPH. This work aimed to evaluate the therapeutic potential of the peripheral CB1 antagonists AM6545 and AM4113 in the treatment of BPH in rats.

In the present study, rats were maintained on a high-salt diet and high-fructose drinking water to create a MetS rat model. The accumulation of visceral fat, increased body weight, insulin resistance, dyslipidemia, and increased mean arterial blood pressure indicated the development of MetS, as previously reported [35]. Most MetS symptoms were mitigated by the CB1 antagonists AM6545 or AM4113. These findings are consistent with our previously published work [25]. Similarly, the peripherally restricted CB1 antagonists JD-5006 and JD-5037 enhanced glucose tolerance and insulin sensitivity [36]. Additionally, these drugs improve fatty liver and dyslipidemia while normalizing body weight and MetS in a mouse model of obesity [37, 38]. The results support the hypothesis that blocking peripheral CB1 receptors is adequate to achieve the most beneficial metabolic effects of globally acting CB1.

Furthermore, BPH was observed along with MetS, as shown by a significant increase in prostate weight. Histological examination of the prostate revealed marked pathological changes in the prostatic ductules marked by a substantial rise in the prostate glandular epithelial height, which is consistent with previous studies [10]. The CB1 antagonists AM6545 and AM4113 prevented these pathological changes in prostate weight and histology. Our findings are consistent with an earlier study that reported that CB1 antagonist help prevent or treat BPH by reducing the size of a noncancerous hypertrophied prostate [16]. The observed effects of CB1 antagonists AM6545 or AM4113 against MetS-induced prostate hyperplasia might be attributed to their antiproliferative properties, as shown by the suppression of cyclin D1 expression. These results are consistent with the known antiproliferative properties of the selective CB1 antagonist rimonabant [39]. Rimonabant has previously been reported to inhibit the proliferation of human peripheral blood mononuclear cells [40] and human breast cancer cells [39] by blocking the Gi/S phase of the cell cycle and decreasing cyclin D expression. The cell cycle blockade induced by CB1 antagonists is due to CB1 receptor inhibition rather than nonspecific effects. Activation of CB1 receptors has been shown to stimulate the PI3K/AKT/mTOR signaling pathway, leading to increased cell proliferation, whereas rimonabant attenuates this signaling pathway resulting in cell cycle arrest [41]. Additionally, the CB1 receptor has been reported to activate the Wnt/β-catenin pathway, which regulates cell cycle progression and proliferation in cancer cells. Inhibition of Wnt/β-catenin pathway by rimonabant was reported in colorectal cancer cells, leading to cell cycle arrest [42]. Therefore, peripheral targeting of CB1 by AM6545 and AM4113 is a promising therapeutic strategy in BPH treatment without the side effects associated with rimonabant that stems from the central CB1 blockade [19].

It has been suggested that oxidative stress has a role in the pathophysiology of MetS [41]. Our observations, consistent with earlier research, demonstrated that MetS exacerbated oxidative stress in the prostate, as shown by the accumulation of MDA and the reduction in the activities of CAT and SOD [42]. Both AM6545 and AM4113 exhibited antioxidant activities, even though AM4113 was significantly more effective in attenuating the increase in MDA content associated with BPH in MetS. Thus, the beneficial effects of AM6545 and AM4113 in this study could be partly due to their significant antioxidant activities. These results are consistent with reports in the literature indicating that CB1 activation is linked to oxidative stress and inflammation [43–45]. The exact signaling pathways involved in the antioxidant activities of AM6545 and AM4113 have not yet been fully elucidated. However, a study by Chang et al. reported that blocking CB1 receptors ammeliorated hepatic inflammation and oxidative stress in a rat model of severely uncontrolled diabetes. These protective effects of rimonabant were mediated through increased activity of the Nrf2-AMPK signaling pathway, a critical regulator of the cellular antioxidant defense shield [43]. Hence, blocking CB1 in prostate tissue would have the potential to alleviate oxidative stress and inflammation in MetS-associated BPH.

Prostatic inflammation is an essential factor that influences the development of BPH, particularly with MetS, because this syndrome is pathologically characterized by systemic inflammation [46]. Previous studies have reported the anti-inflammatory effects of AM6545 and AM4113 in MetS rat models [25, 47]. In the current study, AM6545 and AM4113 exhibited vigorous anti-inflammatory activities, as both agents significantly ameliorated the increase in the expression of the proinflammatory mediators TNF*-α* and IL-6 found in the MetS group. In accordance with these results, blockade of CB1 in adipose and liver tissues was found to attenuate the secretion of inflammatory cytokines and activation of M1 macrophages in rats [48, 49]. Hence, these results confirm that prostatic inflammation could be targeted using CB1 antagonists such as AM6545 and AM4113 as a therapeutic intervention for the BPH treatment.

The ECS has been shown to be dysregulated in many conditions, including obesity and MetS. An increase in 2-AG levels, but not AEA, has been reported in the plasma of patients with MetS [50, 51]. In our recent study, we found an elevation in the endocannabinoid activity in the liver, as indicated by an increase in liver AEA and 2-AG concentrations in the high-fructose/high-salt rat model of MetS [25]. Similarly, an increase in AEA and 2-AG concentrations was observed in the prostate of the MetS-induced BPH rat model. However, AM6545 and AM4113 treatments decreased AEA and 2-AG contents in the prostate. The reduction in AEA and 2-AG levels by CB1 antagonism is proposed due to the negative feedback regulation of endocannabinoid synthesis by CB1 receptor activation. However, when CB1 receptors are blocked, the feedback loop is disrupted, and AEA and 2-AG decrease. This effect has been observed in various settings, including in animal models of obesity and metabolic disorders in addition to clinical trials of CB1 antagonists. The reduction in endocannabinoid levels is believed to mediate the beneficial effects of CB1 antagonism on cardiovascular and metabolic health. In addition, the decrease in endocannabinoid levels may attenuate CB2-mediated signaling in prostatic tissue, where CB2 receptors are expressed and have been shown to regulate prostate growth and inflammation. However, additional research is required to determine the exact contribution of CB2 receptors to the observed effects. Overall, these findings indicate that dysregulation of endocannabinoids in the prostate might contribute to the pathology of MetS-induced BPH, which can be normalized by blocking the CB1 receptor by peripherally acting antagonists.

In addition to the direct inhibition of CB1 receptors in the prostate, it is indeed possible that improvements in blood glucose levels or other metabolic parameters (such as glucose and lipid levels) may have contributed to the observed reduction in BPH. Preclinical studies have demonstrated that inhibition of CB1 receptors can improve metabolic parameters in animal models of MetS [18]. Therefore, it is possible that the observed reduction in BPH in the MetS group may be due to a combination of direct CB1 receptor inhibition in the prostate and improvements in metabolic parameters. Further research is needed to fully elucidate the underlying mechanisms and determine whether CB1 receptor inhibition alone can reduce BPH.

## Conclusion

The CB1 antagonists AM6545 and AM4113 protect against MetS-induced BPH in rats. AM6545 and AM4113 exhibited antiproliferative, antioxidant, and anti-inflammatory effects in the prostate. AM4113 exhibited superior anti-proliferative and antioxidant properties compared to AM6545. This may be attributed to its superior lipid solubility compared to AM6545, which gives it higher permeability to prostate tissue.

## Supplemental Data

**Figure S1.**
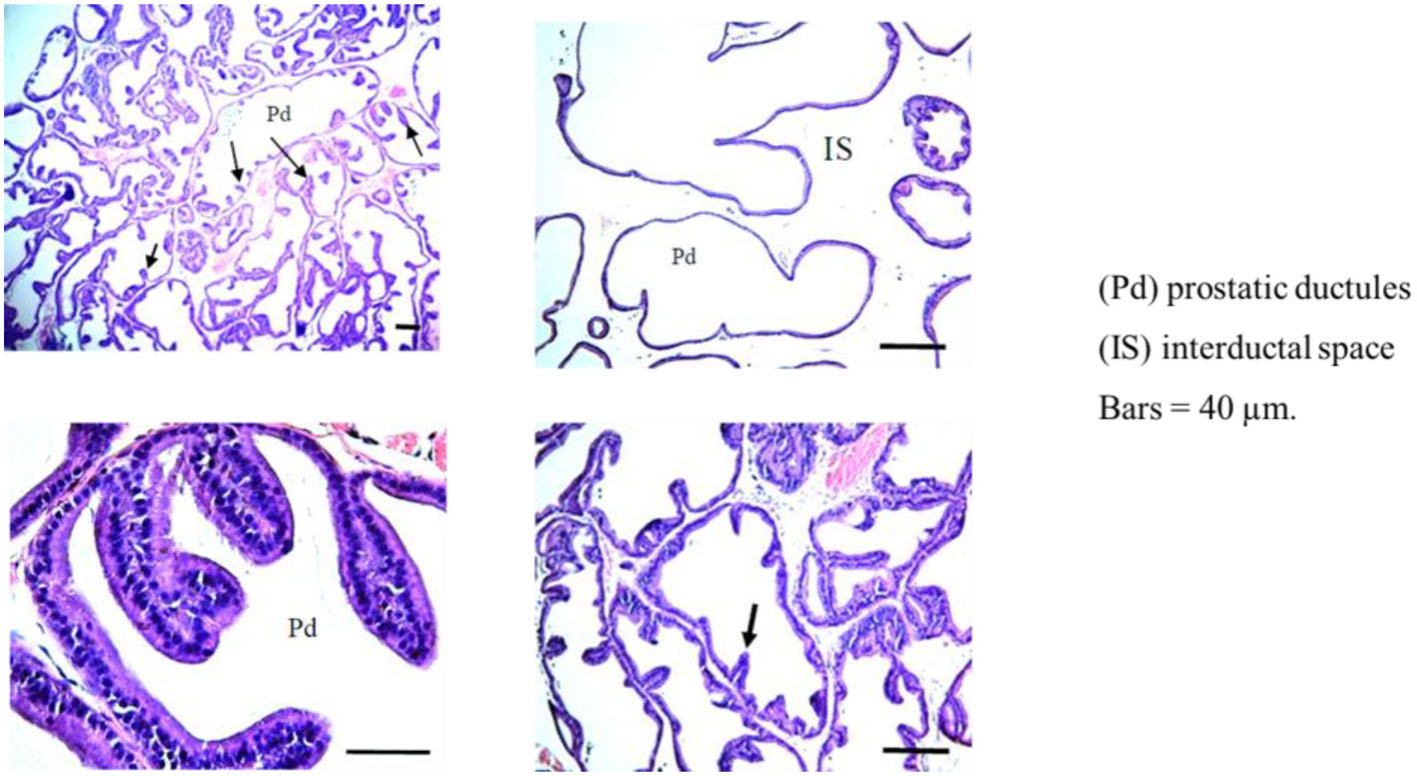
**Metabolic syndrome-induced histopathological changes in rat prostate tissues.** Images were taken from the same group of rats and in some cases from the same animal, but at different magnifications to show the changes in epithelial heights (indicated by arrows).
